# Supplementary material for: Artificial Cathode-Electrolyte Interphase towards High-Performance Lithium-Ion Batteries: A Case Study of β-AgVO3
Source: Nanomaterials (Basel). 2021 Feb 25;11(3):569. doi: 10.3390/nano11030569 (PMC7996271; doi:10.3390/nano11030569)
Supplement: Supplementary file 1 [file nanomaterials-11-00569-s001.pdf]

Supporting Information

Artificial Cathode-electrolyte Interphase towards High-performance Lithium-ion Batteries: A Case Study of β-$AgVO_3$

Liang Liu[1,*], Wei Dai[1], Hongzheng Zhu[2], Yanguang Gu[1], Kangkang Wang[1], Chao Li[1], Chaofeng Pan[1], Min Zhou[3], Jian Liu[2,*]

[1] Automotive Engineering Research Institute, Jiangsu University, Zhenjiang, 212013, China

[2] School of Engineering, Faculty of Applied Science, University of British Columbia, Kelowna, BC V1V 1V7, Canada

[3] Hefei National Laboratory for Physical Sciences at the Microscale, School of Chemistry and Materials Science, University of Science and Technology of China, Hefei, 230026, China

* Correspondence: lliu@ujs.edu.cn (L.L); Jian.liu@ubc.ca (J.L)

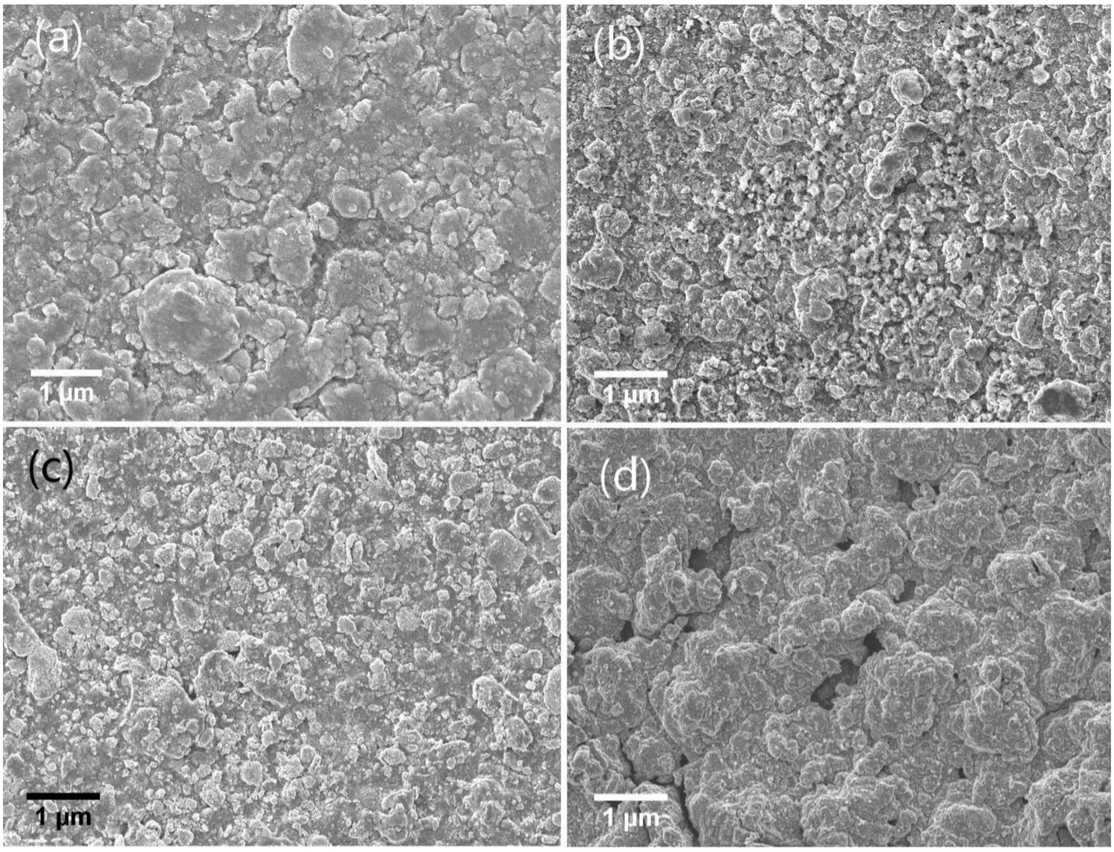

Figure S1. SEM images for different ALD coating layers: (a) SVO-0, (b) SVO-10, (c) SVO-50, (d) SVO-100 after 100 charge-discharge cycles at a current density of 50 mA g^{-1}.

The morphology of prepared SVO electrodes has been examined by SEM after charge-discharge cycles. It is evident that the SVO-0, SVO-10, and SVO-50 (Figure S1a to S1c) have similar morphologies with a lot of small particles on the surface, indicating a more degree of cracking and side reaction. However, the surfaces of the SVO-100 (Figure S1d) look quite different, which exhibits good continuity smooth surfaces with little particles, indicating that the electrode structure becomes more stable with more ALD cycles.

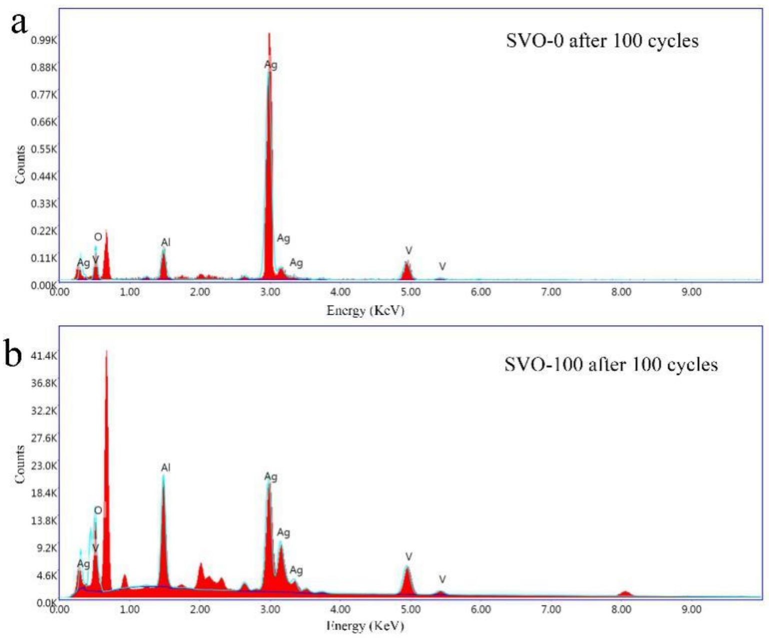

Figure S2 EDX spectra taken from the (a) SVO-0, (b) SVO-100 after 100 charge-discharge cycles.

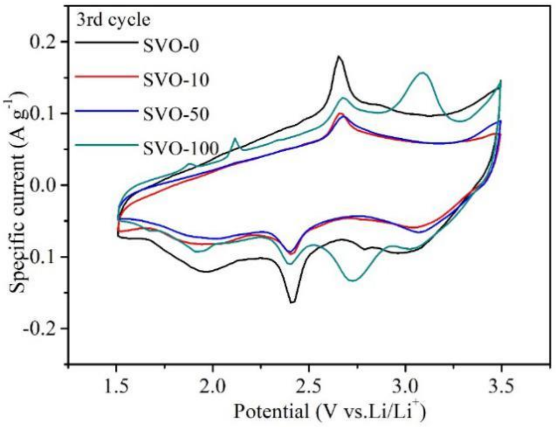

Figure S3. CV curves of (a) SVO-0, (b) SVO-10, (c) SVO-50, (d) SVO-100 at a scanning rate of 0.2 mV s^{-1} in the 3rd cycles.

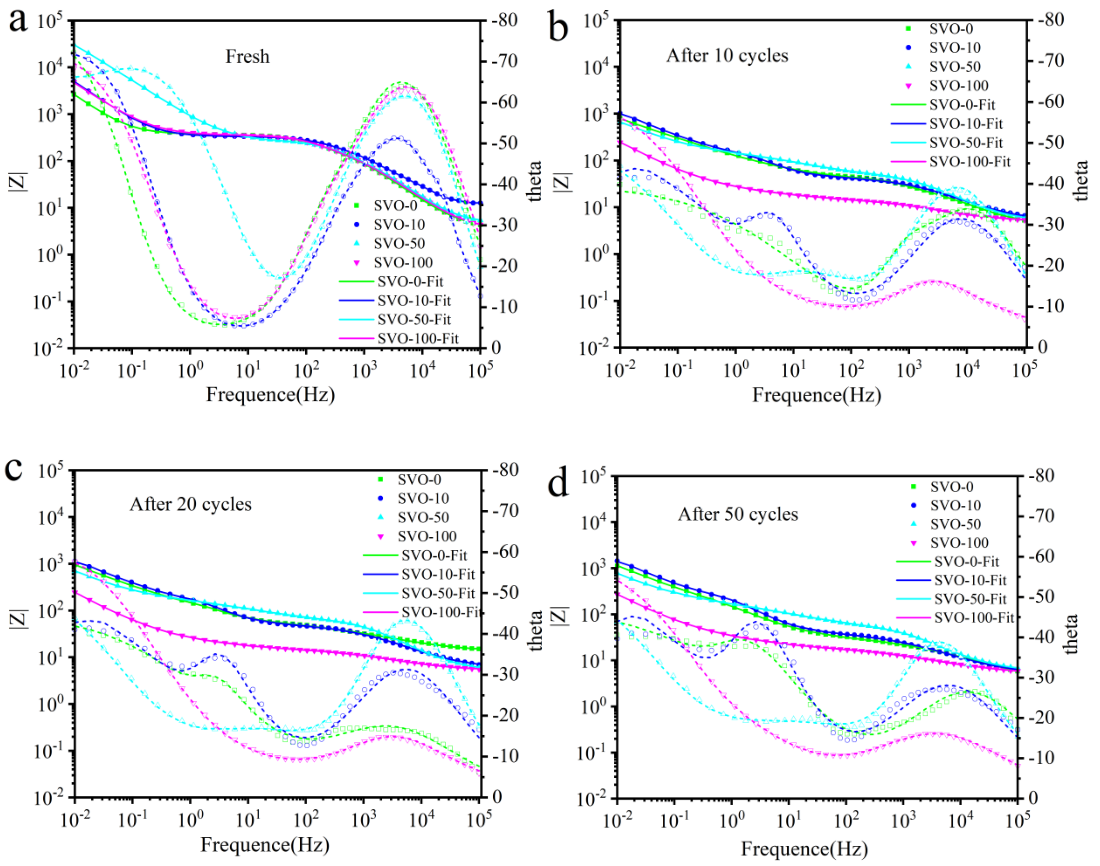

Figure S4. Bode plots for SVO-0, SVO-10, SVO-50, SVO-100 after (a) 0 battery cycle, and (b) 10, (c) 20, (d)50 battery cycles. (The solid line and the solid dot correspond to the |Z|-axis, and the dashed line and the hollow dot correspond to the theta-axis).

Table S1. Values of resistance calculated from fitting the mpedance graphs.

ALD cycle	Ch-disch cycles	$R_s(\Omega)$	Error %	$R_{CEI}(\Omega)$	Error %	$R_{ct}(\Omega)$	Error%	W1-R	Error %	W1-T	Error %	W1-P	Error %
SVO-0	Fresh cell 0 cycle	3.39	1.2	73.39	29.8	258.6	9.8	116.8	16.0	0.48	18.8	0.44	0.5
	After 10 cycles	3.73	3.6	11.91	30.3	17.49	21.3	3787	218.6	604	492.8	0.44	0.7
	After 20 cycles	12.9	1.4	32.42	3.4	20.85	17.9	6196	933.2	767.6	1878.2	0.50	1.3
	After 50 cycles	3.84	4.5	21.67	5.7	53.51	32.9	60641	1.8×10^{-7}	37882	3.5×10^{7}	0.52	2.5
SVO-10	Fresh cell 0 cycle	11.1	1.8	40.91	46.6	280.6	7.1	0.063	3439.9	3.06×10^{-5}	4076.8	0.43	0.4
	After 10 cycles	5.04	2.1	33.93	2.1	41.69	13.2	2567	6.6	93.95	15.4	0.54	1.9
	After 20 cycles	5.70	2.0	38.83	2.7	56.86	14.1	2756	6.0	84.27	14.2	0.55	2.3
	After 50 cycles	4.84	3.4	29.69	5.0	109	17.0	3442	6.6	76.07	15.8	0.58	3.4
SVO-50	Fresh cell 0 cycle	4.75	29.9	36.91	32.1	213.6	6.2	0.02	23824	2.16×10^{-7}	30148	0.40	0.5
	After 10 cycles	4.78	0.89	42.68	2.9	78.64	5.9	2946	40.3	271.5	67.6	0.57	1.3
	After 20 cycles	4.85	1.4	48.93	3.3	109.1	4.9	3078	23.5	247.7	37.4	0.60	1.1
	After 50 cycles	5.04	2.9	33.01	4.8	161.7	5.3	3719	39.9	268.6	63.5	0.62	1.5
SVO-100	Fresh cell 0 cycle	3.48	5.8	63.42	25.6	290.6	6.0	0.016	4528.6	3.58×10^{-6}	5596.5	0.41	0.3
	After 10 cycles	3.55	4.6	54.59	47.7	4.55	5.5	1247	3.9	183.6	5.6	0.70	1.0
	After 20 cycles	4.58	3.9	6.29	6.6	117.7	210.7	1209	3.4	166.7	4.5	0.72	1.8
	After 50 cycles	4.94	0.5	13.1	2.0	12.65	8.1	1307	4.0	188.6	5.4	0.66	0.5
